# Tissue-Specific Browning in Postharvest Litchi Fruit: Comparative Analysis of the Pericarp, Aril, and Seeds

**DOI:** 10.3390/foods15172989

**Published:** 2026-08-25

**Authors:** Sijie Li, Ruihao Zhong, Jinghui Yang, Liyu Fu, Xuequn Pang, Zhaoqi Zhang, Fang Fang

**Affiliations:** 1 College of Horticulture, South China Agricultural University, Guangzhou 510642, China; 18926988953@163.com (S.L.); 15766065756@163.com (J.Y.); lyfu@stu.scau.edu.cn (L.F.); 2 State Key Laboratory for Conservation and Utilization of Subtropical Agro-Bioresources/Guangdong Provincial Key Laboratory of Postharvest Science of Fruit and Vegetables/Engineering Research Center for Postharvest Technology of Horticultural Crops in South China, South China Agricultural University, Guangzhou 510642, China; rhzhong@scau.edu.cn (R.Z.); xqpang@scau.edu.cn (X.P.); 3 College of Life Sciences, South China Agricultural University, Guangzhou 510642, China

**Keywords:** *Litchi chinensis* Sonn., browning mechanism, phenolic metabolism, proanthocyanidins, laccase

## Abstract

Although pericarp browning is a major postharvest challenge in litchi fruit, the browning behavior of the aril and seeds remains poorly understood. This study aimed to investigate tissue-specific browning and the underlying mechanisms. Mature litchi fruit were separated into pericarp, aril, and cut seed, stored at 25 °C. Browning occurred predominantly in the pericarp and seeds, while aril showed no visible browning. The pericarp exhibited greater increases in electrolyte leakage (REL) and malondialdehyde (MDA) content than the aril and seeds. The pericarp and seed were rich in total phenolics, flavonoids, and proanthocyanidins (PAs), all of which declined in both the pericarp and seed during browning, whereas PAs were nearly undetectable in aril. Notably, epicatechin (EC) content was higher in the pericarp than in the seed, while catechin (CT) was abundant in seed but absent in pericarp. Laccase (LAC) and peroxidase (POD) activities, along with transcript levels of *LcLAC14-4/5*, *LcLAC7*, and *LcPOD3*, were substantially higher in the pericarp than in the seed, and barely detectable in aril. The results indicate that tissue-specific browning in litchi fruit is associated with enzymatic oxidation of PA-associated phenolics in the pericarp and seeds, offering a mechanistic target for postharvest preservation. Further studies should explore the biosynthetic regulation of PAs in the pericarp and seeds.

## 1. Introduction

Litchi (*Litchi chinensis* Sonn.) is a commercially valuable fruit of the Sapindaceae family, prized for its distinctive flavor and high economic value, and widely cultivated across subtropical and tropical regions. However, its poor postharvest storability, particularly the rapid pericarp browning that occurs after harvest, severely limits its commercial value [1]. Pericarp browning has long been regarded as a major problem in postharvest handling of litchi fruit. However, whether the aril and seed undergo browning during storage, and how their browning characteristics compare with those of the pericarp, remain unclear.

The peculiar susceptibility of litchi to pericarp browning is intrinsically linked to its unique anatomical structure. Litchi can be regarded as a special type of dry fruit, distinguished by its unique tissue organization [2,3]. Unlike typical fleshy fruits such as peach, where the edible portion derives from the fleshy mesocarp of the ovary wall [4], the edible portion of litchi, namely the fleshy aril, is derived from the funicle rather than the pericarp. Therefore, the pericarp and aril are anatomically distinct tissues, with no direct connection except for their indirect association through the fruit pedicel [5,6,7]. Moreover, the litchi pericarp fully retains its three-layered structure (exocarp, mesocarp, and endocarp), constituting a complete botanical pericarp. However, unlike the fleshy and juicy pericarp of true fleshy fruits, the litchi pericarp is non-succulent, thin, and leathery, with a poorly developed mesocarp layer. These anatomical features render the pericarp highly susceptible to rapid water loss, enzymatic browning, and desiccation immediately after harvest—physiological behaviors that are characteristically observed in dry fruits rather than in fleshy fruits. The litchi aril is surrounded by a pericarp, which acts as a physical barrier that not only separates the aril from the external environment but also serves to protect the internal flesh [8]. Collectively, these structural and postharvest attributes support the classification of litchi as a dry-fruit analogue and provide a fundamental anatomical basis for understanding its distinctive browning phenotype.

Considerable progress has been made in elucidating the mechanisms underlying pericarp browning. Litchi pericarp browning is closely associated with water loss and enzymatic oxidation. It is well established that postharvest water loss triggers pericarp color deterioration, elevates the browning index, and increases membrane permeability [9,10], whereas maintaining water supply effectively reduces water loss and delays browning [11]. During postharvest storage, water loss and membrane disruption in the pericarp may impair cellular compartmentalization, thereby increasing the contact between phenolic substrates and oxidative enzymes and promoting enzymatic browning reactions [9,11]. At the biochemical level, pericarp browning is associated with enzymatic oxidation, accompanied by both anthocyanin degradation and phenolic oxidation. Previous studies have shown that the anthocyanin degradation-related enzyme in litchi pericarp is essentially a laccase, which can promote anthocyanin degradation in the presence of epicatechin and lead to the formation of brown polymeric products [12,13]. Further evidence indicates that LAC-mediated oxidative polymerization of flavonoids is a major browning pathway, and the postharvest depletion of phenolic and flavonoid substrates closely correlates with the accumulation of brown polymers [14,15]. Moreover, *LcLAC14-4* and other members of the laccase gene family have been shown to participate in polyphenol metabolism and tissue browning in litchi [16,17].

Despite these advances, studies have focused almost exclusively on the pericarp, and the browning behavior of the aril and seeds has received little attention. An exclusive focus on the pericarp risks missing critical clues regarding inter-tissue interactions. With prolonged storage, pericarp water loss and oxidative stress can affect aril quality, manifesting as increased translucency, darker coloration, and flavor deterioration in the aril [1]. Nevertheless, systematic comparisons of browning behavior and its physiological basis across the pericarp, aril, and seeds remain lacking, particularly regarding whether they share similar phenolic substrate profiles and oxidative enzyme systems.

In order to investigate whether the aril and seed share phenolic substrates and oxidative enzyme systems analogous to those of the pericarp, this study employed postharvest mature litchi fruit for a comparative analysis of browning behavior and associated physiological and biochemical alterations across the three tissues during storage. We determined browning indices, color parameters, membrane damage indicators, phenolic composition, and the activities and gene expression of browning-related enzymes (LAC/POD), with the aim of deciphering tissue-specific variations in browning occurrence, substrate accumulation, and mechanistic pathways. The findings provided a tissue-specific physiological basis of postharvest browning in litchi fruit, thereby informing targeted preservation strategies to effectively retard pericarp browning.

## 2. Materials and Methods

### 2.1. Plant Materials

**Figure 1:**
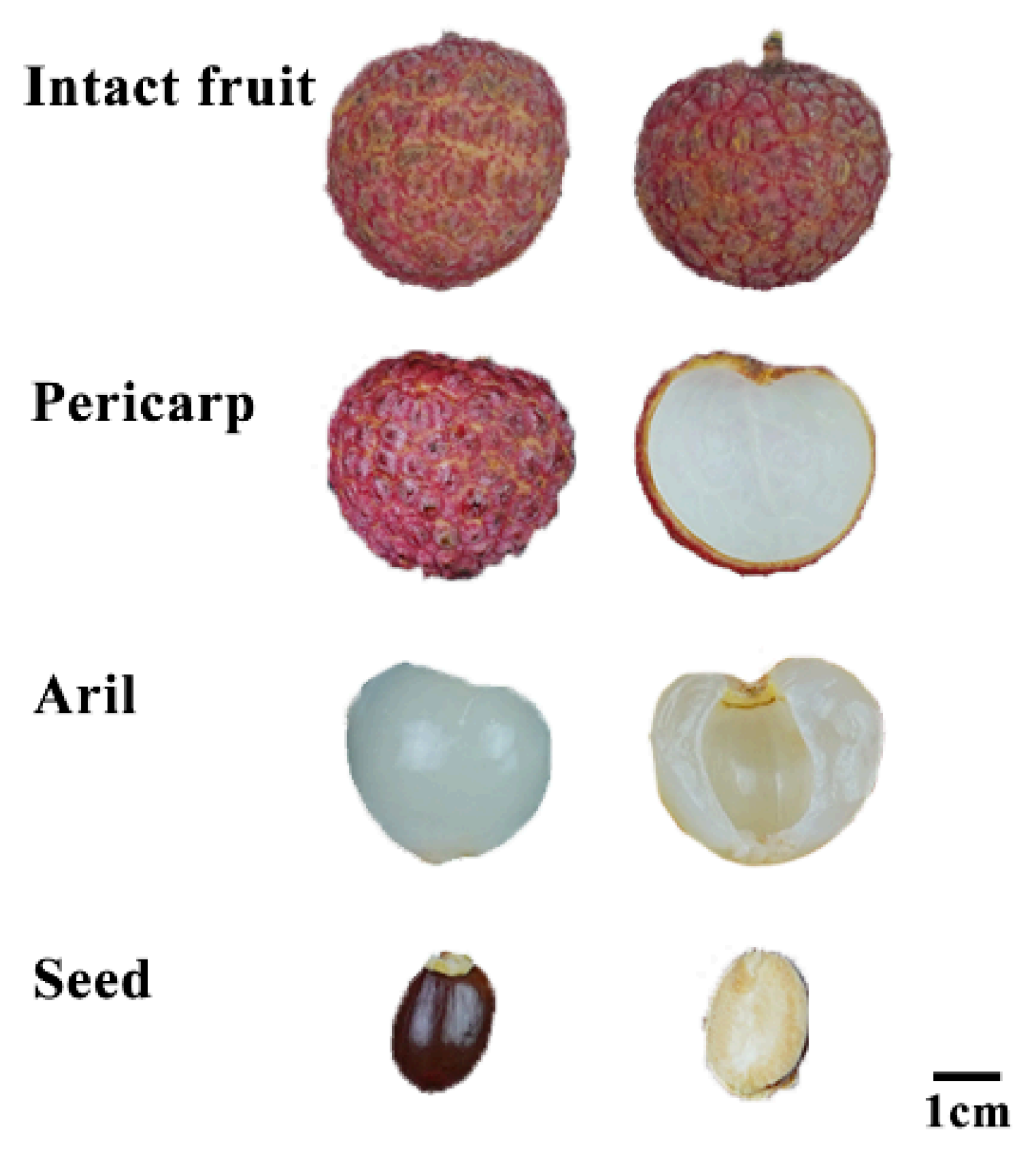
Representative photographs of the intact litchi fruit and the separated pericarp, aril, and seed. Scale bar = 1 cm.

Mature litchi fruit (*Litchi chinensis* Sonn. cv. ‘Huaizhi’) at similar stages with red appearance (around 85 days after anthesis) were harvested from an orchard in Conghua, Guangzhou, Guangdong, China. Litchi trees in the orchard aged over 30 years were cultivated in organic-rich soils with good drainage. After harvest, the fruit were placed in a foam box with ice packs at the bottom, separated by a layer of absorbent paper, and immediately transported to the laboratory within approximately one hour. Fruit with uniform size and color and free of visible diseases and insect damage were selected for the experiment. The pericarp, aril, and seeds were manually separated using the same stainless steel knife and cut into halves (Figure 1). The separated tissues were stored unpackaged in a constant-temperature incubator at 25 °C and sampled at seven time points: 0, 12, 24, 36, 48, 60, and 72 h. A total of 210 fruits were randomly divided into seven storage time points with three biological replicates of 10 fruits each at each time point. For each replicate, the same tissue (pericarp, aril, or seeds) from all 10 fruits was pooled into one biological sample, giving three independent biological replicates per tissue per time point (*n* = 3). After sampling, all tissues were frozen in liquid nitrogen and stored at −80 °C until further analysis.

### 2.2. Determination of Browning Index and Color Parameters

The browning grade of the pericarp, aril, and seed was determined according to the method described by Liu et al. [2] The browning index was calculated using the following formula:

Browning index = Σ (browning grade × number of fruit at each grade)/total number of samples

The L*, a*, and b* values of different litchi tissues during storage were measured using a Minolta CR-300 colorimeter (Konica Minolta Co., Ltd., Osaka, Japan). The total color difference (ΔE) was calculated according to the following equation:

$$\Delta E\;=\;\sqrt{\left\{{\left(L_{t}^{*}-\;L_{0}^{*}\right)}^{2}+\;{\left(a_{t}^{*}-\;a_{0}^{*}\right)}^{2}+\;{\left(b_{t}^{*}-\;b_{0}^{*}\right)}^{2}\right\}}$$

where L_0_*, a_0_*, and b_0_* are the initial color values at 0 h, and L_t_*, a_t_*, and b_t_* are the corresponding color values at each storage time.

### 2.3. Determination of Anthocyanin Content

Anthocyanin content was determined using the pH differential method described by Fang et al. [13], with slight modifications. Briefly, 0.10 g of powdered tissue from different litchi tissues was extracted with 1.0 mL of 0.5% HCl solution by ultrasonication for 20 min, followed by centrifugation to collect the supernatant. The residue was re-extracted with 0.50 mL of the same extraction solution, and the resulting supernatants were combined. An aliquot of 0.25 mL of the extract was separately diluted to 1.25 mL with 0.4 mol·L^−1^ KCl–HCl buffer (pH 1.0) or 0.4 mol·L^−1^ citric acid–disodium hydrogen phosphate buffer (pH 5.0). After thorough mixing, the absorbance was measured at 510 nm using a spectrophotometer (Multiskan Sky, Thermo Fisher Scientific, Waltham, MA, USA). Three biological replicates were performed for each treatment. Anthocyanin content (mg g^−1^ FW) = ΔA × 445.2 × 5 × 15/(29,600 × 1), where ΔA = A_510_ (pH 1.0) − A_510_ (pH 5.0).

### 2.4. Determination of Relative Electrolyte Leakage and Malondialdehyde Content

Relative electrolyte leakage (REL) was determined according to the method of Huang et al. [18], with slight modifications. Fresh samples (1.00 g) from each tissue were immersed in 20 mL of distilled water for 30 min, and the initial electrical conductivity (R_1_) was measured using an Aquasearcher AB33EC benchtop conductivity meter (OHAUS Corporation, NJ, USA). The samples were then boiled for 15 min and cooled to room temperature, after which the final electrical conductivity was measured as R_2_. Relative electrolyte leakage was calculated as R_1_/R_2_ × 100%.

Malondialdehyde (MDA) content was determined using the thiobarbituric acid (TBA) method [19] with slight modifications. Briefly, 0.10 g of frozen litchi tissue powder was homogenized with 1.0 mL of pre-cooled 0.05 M PBS containing 0.05 g PVP and centrifuged at 15,000 rpm for 20 min at 4 °C. The supernatant was mixed with an equal volume of 20% trichloroacetic acid containing 0.5% TBA and heated in a boiling water bath for 20 min. After rapid cooling and centrifugation at 4000 rpm for 20 min, the absorbance was measured at 450, 532, and 600 nm using a spectrophotometer (Multiskan Sky, Thermo Fisher Scientific, Waltham, MA, USA). MDA content was calculated as [6.45 × (A_532_ − A_600_) − 0.56 × A_450_] × 10, and the results were expressed as nmol g^−1^ FW.

### 2.5. Determination of Total Phenolic and Total Flavonoid Contents

Total phenolic content was determined using the Folin–Ciocalteu method described by Singleton et al. [20], with slight modifications. Briefly, 0.10 g of powdered tissue from different litchi tissues was extracted with 1.0 mL of 60% ethanol in an ice bath for 30 min, followed by centrifugation. An aliquot of 0.05 mL of the supernatant was mixed with 0.10 mL of Folin–Ciocalteu reagent and incubated for 2 min. Subsequently, 0.50 mL of 1 mol·L^−1^ sodium carbonate solution and 0.35 mL of distilled water were added. After incubation in the dark for 60 min, absorbance was measured at 765 nm using a spectrophotometer (Multiskan Sky, Thermo Fisher Scientific, Waltham, MA, USA). Gallic acid was used to construct the standard curve (y = 1.1629x + 0.0477, *R*^2^ = 0.9996), and total phenolic content was expressed as milligrams of gallic acid equivalents per gram of fresh weight (mg GAE g^−1^ FW).

Total flavonoid content was determined using a modified method of Jia et al. [21]. Briefly, 10 μL aliquot of the above ethanolic extract was mixed with 190 μL of 60% ethanol. and 50 μL of 5% NaNO_2_ solution. After incubation for 6 min, 50 μL of 10% Al(NO_3_)_3_ solution was added, followed by another 6 min of incubation. Subsequently, 400 μL of 4% NaOH solution was added, and the mixture was incubated for 10 min. Absorbance was measured at 510 nm using 60% ethanol as the blank. Rutin was used to construct the standard curve (y = 0.914x − 0.0003, *R*^2^ = 0.9992), and total flavonoid content was expressed as milligrams of rutin equivalents per gram of fresh weight (mg RE g^−1^ FW).

### 2.6. Determination of Laccase and Peroxidase Activities

Laccase (LAC) activity was determined according to the method of Fang et al. [13] with appropriate modifications. Epicatechin at a concentration of 2 mM was used as the substrate. The substrate solution and enzyme extract were mixed at a ratio of 1:1, and the change in absorbance at 380 nm was immediately monitored continuously for 3 min. Enzyme extract inactivated by boiling water bath treatment was used as the control. One unit of LAC activity was defined as a 0.01 increase in OD_380_ per minute per gram of fresh tissue, and the results were expressed as U g^−1^ FW.

Peroxidase (POD) activity was assayed with slight modifications according to Mizobutsi et al. [17]. Frozen litchi tissue powder was extracted with pre-cooled phosphate buffer, and the supernatant was used as the crude enzyme extract. POD activity was determined by monitoring the increase in absorbance at 470 nm in a reaction mixture containing phosphate buffer, guaiacol, and H_2_O_2_. One unit of POD activity was defined as a 0.005 increase in OD_470_ per minute per gram of fresh tissue, and the results were expressed as U g^−1^ FW.

### 2.7. Quantitative Analysis of Gene Expression by qRT-PCR

Total RNA was extracted from the samples using a Fast RNA Extraction Kit 3.0 (Beijing Huayueyang Biotechnology Co., Ltd., China). After RNA concentration and purity were assessed, first-strand cDNA was synthesized using the PrimeScript™ RT reagent Kit with gDNA Eraser (Perfect Real Time; Takara, Japan), which was also used to remove residual genomic DNA contamination. qRT-PCR analysis was then performed using SYBR Green dye on a CFX96 Real-Time PCR Detection System (Bio-Rad, Hercules, CA, USA). Litchi Actin (LITCHI007623) was used as the internal reference gene. Melting curves and standard curves were analyzed using CFX Manager Software version 3.1 (Bio-Rad, Hercules, CA, USA). to ensure amplification specificity and efficiency. The PCR program was as follows: 95 °C for 3 min, followed by 40 cycles of 95 °C for 15 s, 56 °C for 30 s, and 72 °C for 35 s. The relative expression levels of target genes were calculated using the 2^−ΔΔCt^ method. The primer sequences used in this study are listed in Supplemental Table S1.

### 2.8. HPLC Analysis of Different Tissues of Litchi Fruit

HPLC analysis of flavonoids in different litchi tissues was performed according to the method of Lv et al. [22] with slight modifications. Samples from each tissue were vacuum freeze-dried and ground into powder and 0.1 g of each sample was defatted with 1.0 mL of n-hexane containing 3% (*v*/*v*) formic acid and 1% (*w*/*v*) BHT. After vortexing for 5 min, the mixture was centrifuged at 10,000× *g* for 10 min at 4 °C. The supernatant was discarded, and the residue was dried under reduced pressure at low temperature for approximately 1 h. Flavonoids were then extracted three times, each with 1.0 mL of methanol containing 3% (*v*/*v*) formic acid and 1% (*w*/*v*) BHT. For each extraction, the mixture was vortexed for 5 min, ultrasonicated at 200 W for 30 min at 4 °C, and centrifuged at 10,000× *g* for 10 min at 4 °C. The supernatants obtained from the three extractions were combined and filtered through a 0.22 μm membrane filter before HPLC analysis.

The samples were analyzed using an Agilent 1260 HPLC system (Agilent Technologies, Santa Clara, CA, USA) equipped with a ZORBAX Eclipse XDB C18 column (5 μm, 4.6 × 150 mm). The column temperature was maintained at 30 °C, with a flow rate of 0.5 mL/min and an injection volume of 10 μL. Methanol (solvent A) and 0.2% (*v*/*v*) formic acid in water (solvent B) were used as the mobile phases. The gradient program was as follows: 0–15 min, 17–21% A; 15–28 min, 21% A; 28–32 min, 21–26% A; 32–45 min, 26–27% A; 45–60 min, 27–31% A; 60–70 min, 31–53% A; 70–80 min, 53–90% A; 80–85 min, 90–17% A; and 85–90 min, 17% A. All analytes were detected at 280 nm. Epicatechin (EC), catechin (CT), procyanidin A2 (PC A2), procyanidin B1 (PC B1), and procyanidin B2 (PC B2) were identified by comparing their retention times with those of authentic standards under the same conditions. The retention times of EC, CT, PC A2, PC B1, and PC B2 were approximately 38.47, 18.87, 62.17, 12.00, and 22.70 min, respectively. Quantification was performed using the external-standard method, and the contents of each compound were calculated from the corresponding calibration curves.

### 2.9. Statistical Analysis

All data are presented as the mean ± standard error (SE) of three biological replicates. For variables measured in the three tissues throughout storage, two-way analysis of variance (ANOVA) was used to evaluate the effects of tissue type (pericarp, aril, and seed), storage time (0, 12, 24, 36, 48, 60, and 72 h), and their interaction. Differences among the three tissues at each storage time were further analyzed using one-way ANOVA followed by Duncan’s multiple range test at *p* < 0.05. Statistical analyses were performed using IBM SPSS Statistics, version 22.0 (IBM Corp., Armonk, NY, USA). Graphs were generated using Origin 2021 (OriginLab Corp., Northampton, MA, USA).

## 3. Results

### 3.1. Appearance and Browning Phenomena in Different Tissues During Storage

**Figure 2:**
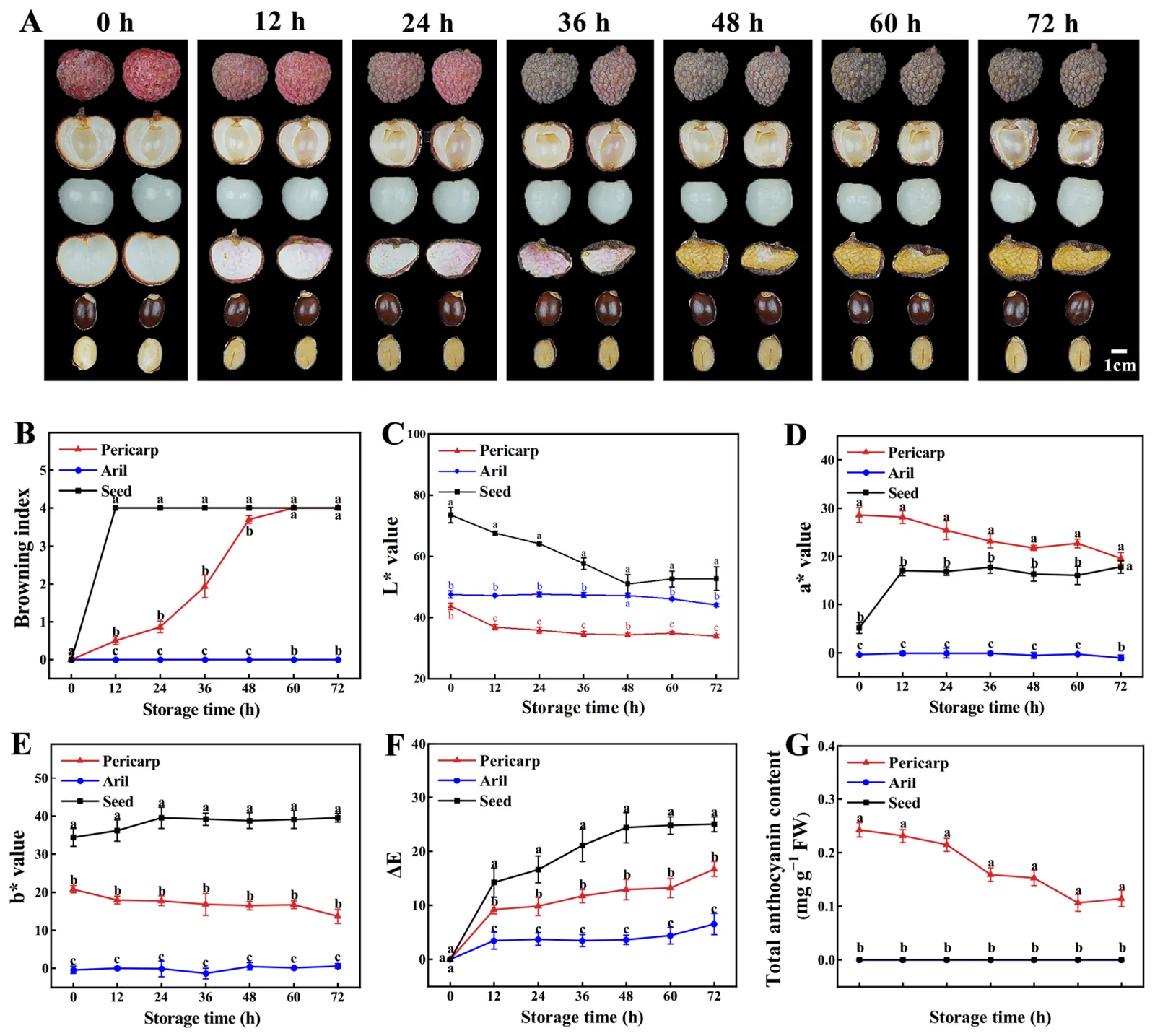
Browning phenotype and color parameters in different tissues of litchi fruit during storage. Visual appearance changes (**A**), browning index (**B**), L* values (**C**), a* values (**D**), b* values (**E**), ΔE value (**F**), total anthocyanin content (**G**). Data are means ± standard error (SE), *n* = 3. Different lowercase letters (a, b, c) indicate significant differences among tissues at the same storage time (*p* < 0.05).

To investigate tissue-specific browning, mature litchi fruit were separated into pericarp, aril, and seeds, stored at 25 °C for 72 h, and differences in appearance were observed among the three tissues (Figure 2A). During storage, pericarp browning gradually intensified, with the surface color changing from bright red to dark brown. The cut surface of the seeds browned rapidly, whereas no obvious change was observed in the aril throughout the storage period. The browning index of the pericarp increased significantly with storage time (*p* < 0.05) and reached the highest level between 48 and 72 h. The browning index of the exposed cut surface of the seeds increased rapidly at the 12 h time point, reaching a value of 4.0. In contrast, the browning index of the aril maintained a persistently low level, close to 0 throughout storage (Figure 2B). The L* values of the seeds and pericarp tended to decrease, while those of the aril remained largely unchanged during storage (Figure 2C). The a* value of the pericarp was the highest, and it gradually decreased with storage time. The a* value of the seed increased rapidly and then remained relatively stable after 12 h, whereas that of the aril was close to 0 (Figure 2D). The b* values were highest and relatively stable in the seed, whereas they progressively declined in the pericarp and remained consistently low in the aril (Figure 2E). Consistently, the ΔE value increased continuously in both the pericarp and seeds during storage, indicating pronounced color changes in these two tissues, while the aril maintained a much lower ΔE value throughout storage (Figure 2F). Total anthocyanin content was detected only in the pericarp and gradually decreased during storage, reaching 47.37% of its initial level at 72 h. Anthocyanins were not detected in the aril or seeds throughout storage (Figure 2G). In summary, postharvest browning in litchi fruit exhibited tissue specificity. The seed browned rapidly immediately after cutting, the pericarp underwent progressive browning over storage time, whereas the aril remained largely stable throughout.

Two-way ANOVA revealed that tissue type significantly affected all analyzed variables (*p* < 0.01; Table 1). Storage time also exerted significant effects on all variables except the b* value. Significant tissue × storage time interactions were observed for all variables except *LcLAC14-4* expression, indicating that the temporal responses of most physiological, biochemical, and molecular characteristics differed among the pericarp, aril, and seeds. To further explore these interactions, one-way ANOVA followed by Duncan’s multiple range test was performed to compare tissue types at each storage time.

### 3.2. Differences in Membrane Integrity Among the Aril, Pericarp, and Seeds

**Figure 3:**
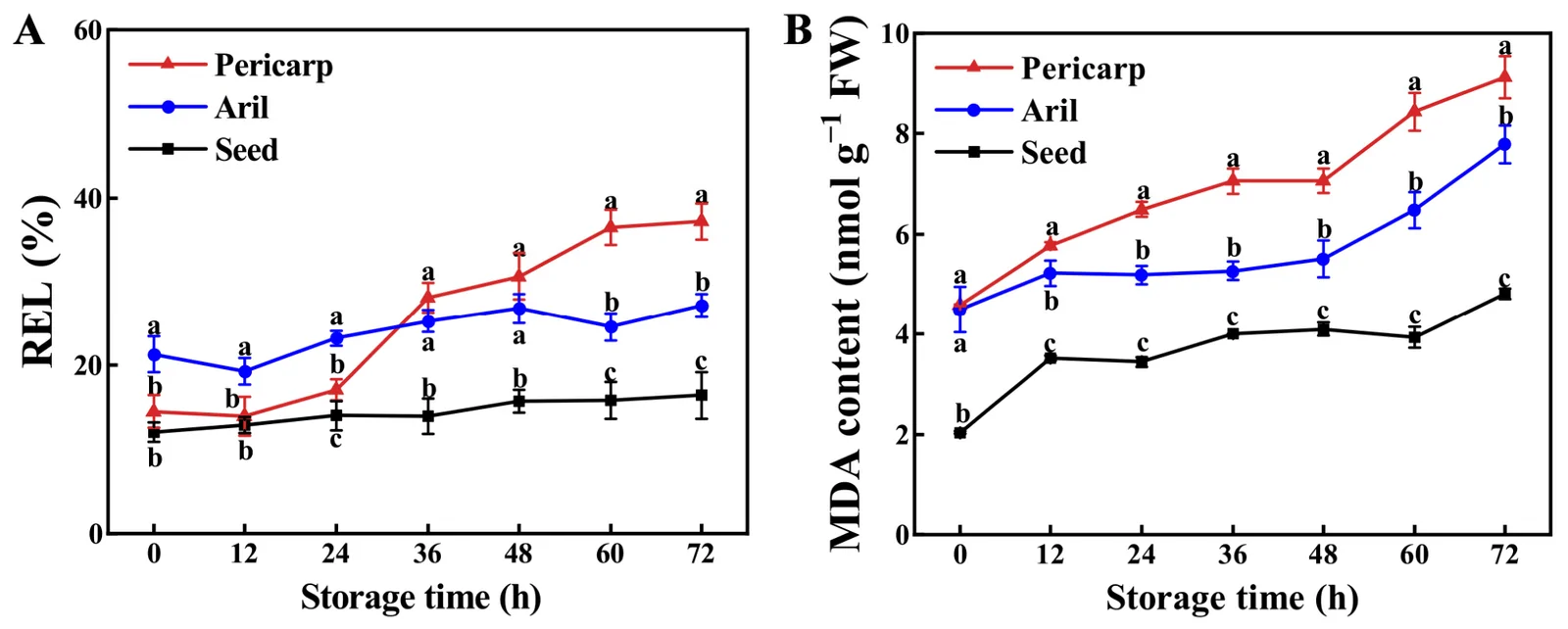
Changes in membrane integrity in different tissues of litchi fruit during storage. (**A**) REL, (**B**) MDA. Data are means ± SE, *n* = 3. Different lowercase letters indicate significant differences among tissues at the same storage time (*p* < 0.05).

Relative electrolyte leakage (REL) increased in all tissues during storage, with the pericarp exhibiting the most pronounced increase (Figure 3A). The REL values of the pericarp, aril, and seeds all exhibited an upward trend throughout the storage period. By 72 h of storage, REL in the pericarp had risen to approximately 156% relative to 0 h, while the corresponding increases in aril and seeds were approximately 27% and 35%, respectively. Malondialdehyde (MDA) content increased progressively in all tissues, with the highest levels consistently observed in the pericarp and the lowest in the seed (Figure 3B). By 72 h, MDA content had risen by approximately 100%, 73%, and 136% in the pericarp, aril, and seeds, respectively, compared with their initial levels. Collectively, these findings demonstrate that membrane integrity was compromised in all tissues, with the pericarp being the most severely affected.

### 3.3. Total Phenolic and Total Flavonoid Contents in Different Tissues of Litchi Fruit During Storage

**Figure 4:**
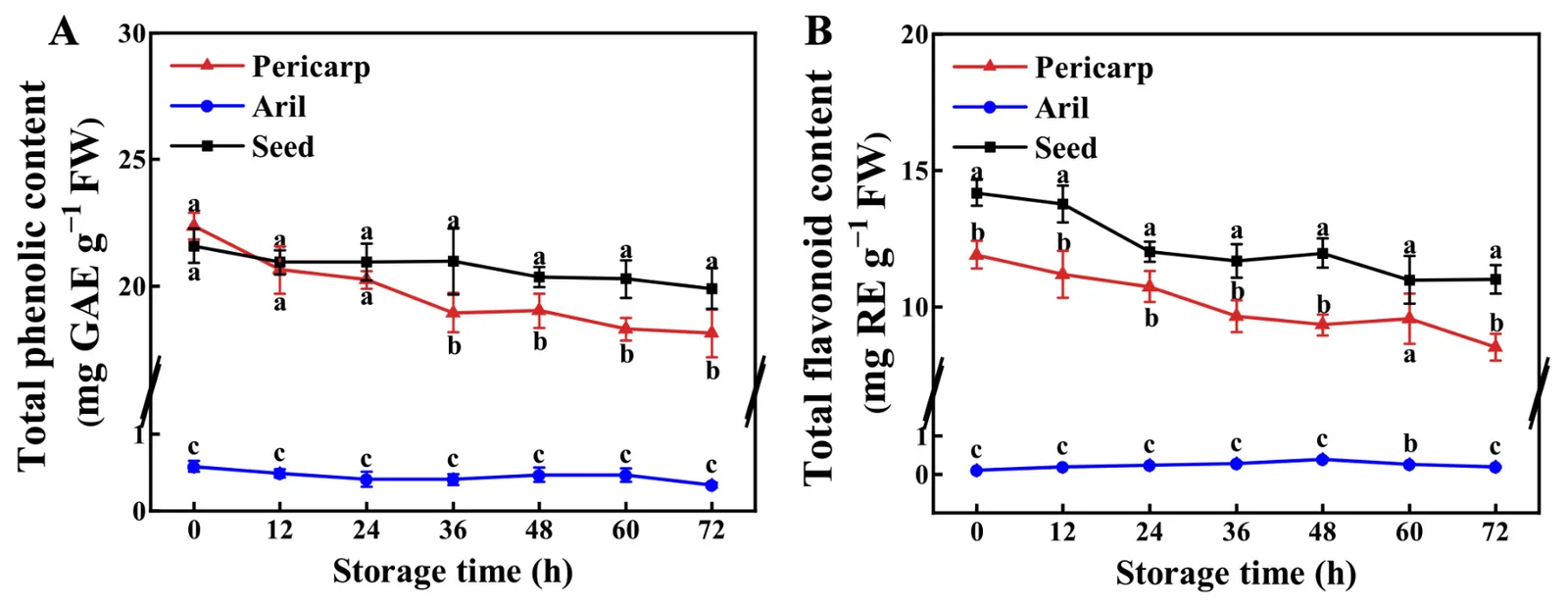
Changes in total phenolic and total flavonoid contents in different tissues of litchi fruit during storage. (**A**) Total phenolic content, (**B**) total flavonoid content. Data are means ± SE, *n* = 3. Different lowercase letters indicate significant differences among tissues at the same storage time (*p* < 0.05).

Total phenolic content declined in all three tissues during storage; however, it remained relatively high in the pericarp and seeds, while staying at a consistently low level in the aril (Figure 4A). In the pericarp, total phenolic content gradually decreased from approximately 22.3 mg GAE g^−1^ FW at 0 h to 18.1 mg GAE g^−1^ FW at 72 h, corresponding to about 81.2% of the initial level. In contrast, the seed maintained a relatively stable total phenolic content, decreasing slightly from approximately 21.6 mg GAE g^−1^ FW at 0 h to 19.9 mg GAE g^−1^ FW at 72 h (∼92.1% of the initial level). The aril consistently exhibited significantly lower levels (*p* < 0.05), declining from approximately 0.58 mg GAE g^−1^ FW at 0 h to 0.33 mg GAE g^−1^ FW at 72 h. Similarly, the total flavonoid content in the pericarp and seeds was high but showed a decreasing trend, whereas that in the aril remained consistently at a very low level (Figure 4B). In the pericarp, total flavonoid content decreased from approximately 12.0 mg RE g^−1^ FW at 0 h to 8.6 mg RE g^−1^ FW at 72 h, representing about 72% of the initial value. Seed total flavonoid content declined from approximately 14.2 to 11.0 mg RE g^−1^ FW (∼77.5% of the initial level), whereas the aril remained consistently low throughout storage. Taken together, these results demonstrate that total phenolic and total flavonoid contents exhibited pronounced tissue-specific distribution patterns, with significantly higher levels in the pericarp and seeds than in the aril (*p* < 0.05), and a greater decrease observed in the pericarp compared with the seeds.

### 3.4. Comparative Proanthocyanidin Composition Among Aril, Pericarp, and Seeds

**Figure 5:**
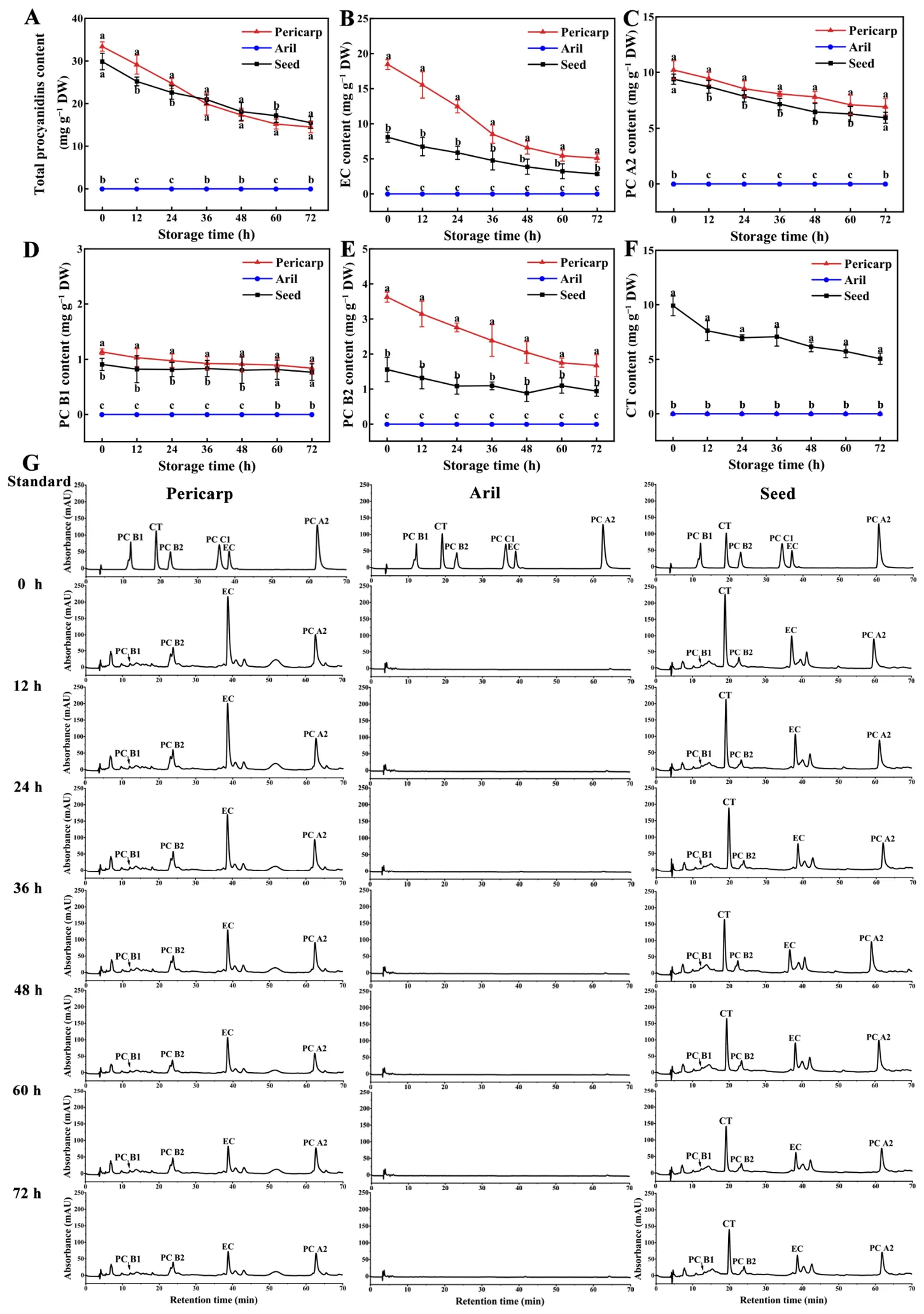
HPLC analysis and content changes in PAs in different tissues of litchi fruit during storage. Changes in the contents of total PA (**A**), EC (**B**), PC A2 (**C**), PC B1 (**D**), PC B2 (**E**), and CT (**F**) in the three tissues. (**G**) Representative HPLC chromatograms of the three tissues. Data are means ± SE, *n* = 3. Different lowercase letters indicate significant differences among tissues at the same storage time (*p* < 0.05).

HPLC analysis was conducted to characterize the major proanthocyanidin (PA) compounds in different tissues of litchi fruit during storage (Figure 5). The pericarp and seeds exhibited qualitatively similar PA profiles, with epicatechin (EC), PC A2, PC B2, and PC B1 identified as the predominant components in both tissues, but at markedly different concentrations—EC being the most abundant and PC B1 the least. All PAs declined progressively throughout storage, with the pericarp showing consistently greater decreases than the seeds, whereas no PAs were detectable in the aril. Specifically, the pericarp contained relatively high levels of total PA, which decreased from approximately 33.4 mg g^−1^ DW at 0 h to 14.5 mg g^−1^ DW at 72 h (∼57% reduction), whereas that in the seed declined from approximately 20.0 mg g^−1^ DW at 0 h to 10.5 mg g^−1^ DW at 72 h (∼48% reduction) (Figure 5A). EC, the predominant compound, decreased from approximately 18.4 mg g^−1^ DW at 0 h to 5.1 mg g^−1^ DW at 72 h in the pericarp and from 8.1 mg g^−1^ DW at 0 h to 2.8 mg g^−1^ DW at 72 h in the seeds, corresponding to reductions of approximately 72% and 65%, respectively (Figure 5B). PC A2 content decreased from approximately 10.2 to 6.9 mg g^−1^ DW in the pericarp and from 9.4 to 5.9 mg g^−1^ DW in the seeds between 0 and 72 h, corresponding to reductions of approximately 32% and 37%, respectively (Figure 5C). PC B1 content declined from approximately 1.1 mg g^−1^ DW at 0 h to 0.8 mg g^−1^ DW at 72 h in the pericarp and from 0.9 mg g^−1^ DW at 0 h to 0.8 mg g^−1^ DW at 72 h in the seed, representing decreases of approximately 27% and 11%, respectively (Figure 5D). PC B2 content decreased from approximately 3.1 mg g^−1^ DW at 0 h to 1.7 mg g^−1^ DW at 72 h in the pericarp and from 1.3 mg g^−1^ DW at 0 h to 0.9 mg g^−1^ DW at 72 h in the seeds, corresponding to reductions of approximately 45% and 31%, respectively (Figure 5E). In addition, catechin (CT) was detected mainly in the seed (Figure 5F). The CT content in the seeds decreased continuously during storage, from approximately 9.9 mg g^−1^ DW at 0 h to 5.1 mg g^−1^ DW at 72 h, representing a reduction of approximately 43%. However, CT was almost undetectable in the pericarp and aril. By contrast, none of these PA compounds were detected in the aril (Figure 5G). Overall, the pericarp and seeds had similar PA compositions but differed significantly in content and the degree of decrease (*p* < 0.05), with the pericarp showing higher initial levels and larger proportional declines, while PAs were undetectable in the aril.

### 3.5. Differences in LAC and POD Activities Among the Aril, Pericarp, and Seeds

**Figure 6:**
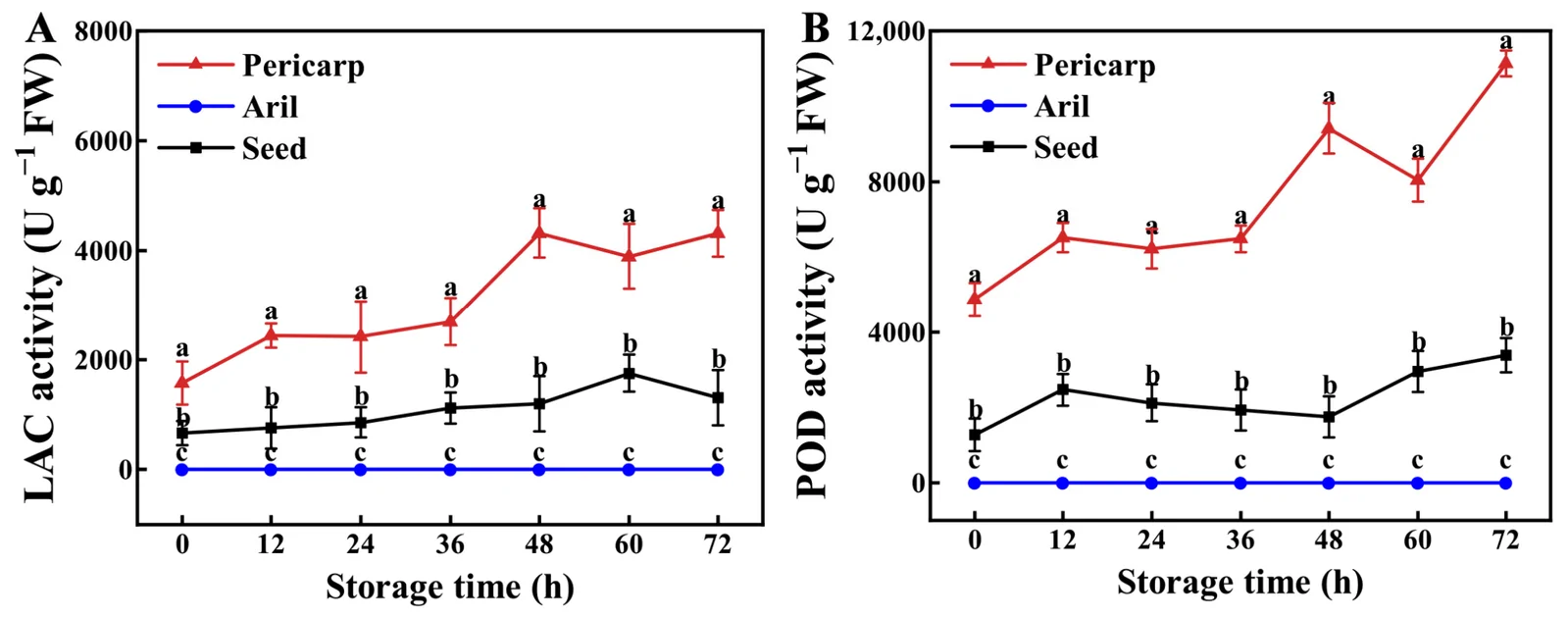
Changes in oxidative enzyme activities in different tissues of litchi fruit during postharvest storage. (**A**) LAC activity, (**B**) POD activity. Data are means ± SE, *n* = 3. Different lowercase letters indicate significant differences among tissues at the same storage time (*p* < 0.05).

LAC and POD activities exhibited clear tissue-specific differences during storage, with the highest activities observed in the pericarp, followed by the seeds, while both enzymes were undetectable in the aril (Figure 6). Pericarp LAC activity increased from approximately 1580 U g^−1^ FW at 0 h to 4312 U g^−1^ FW at 72 h, representing an increase of about 173%, whereas seeds LAC activity increased from approximately 667 U g^−1^ FW at 0 h to 1317 U g^−1^ FW at 72 h, an increase of about 86% (Figure 6A). Similarly, pericarp POD activity increased from approximately 4885 U g^−1^ FW at 0 h to 11,134 U g^−1^ FW at 72 h, corresponding to an increase of about 128%, while seeds POD activity increased from approximately 1272 U g^−1^ FW at 0 h to 3377 U g^−1^ FW at 72 h, an increase of about 165% (Figure 6B). Despite the relatively large proportional increase in the seed, the absolute activities of both enzymes remained markedly higher in the pericarp throughout storage.

### 3.6. Differences in LAC and POD Gene Expression Among the Aril, Pericarp, and Seeds

**Figure 7:**
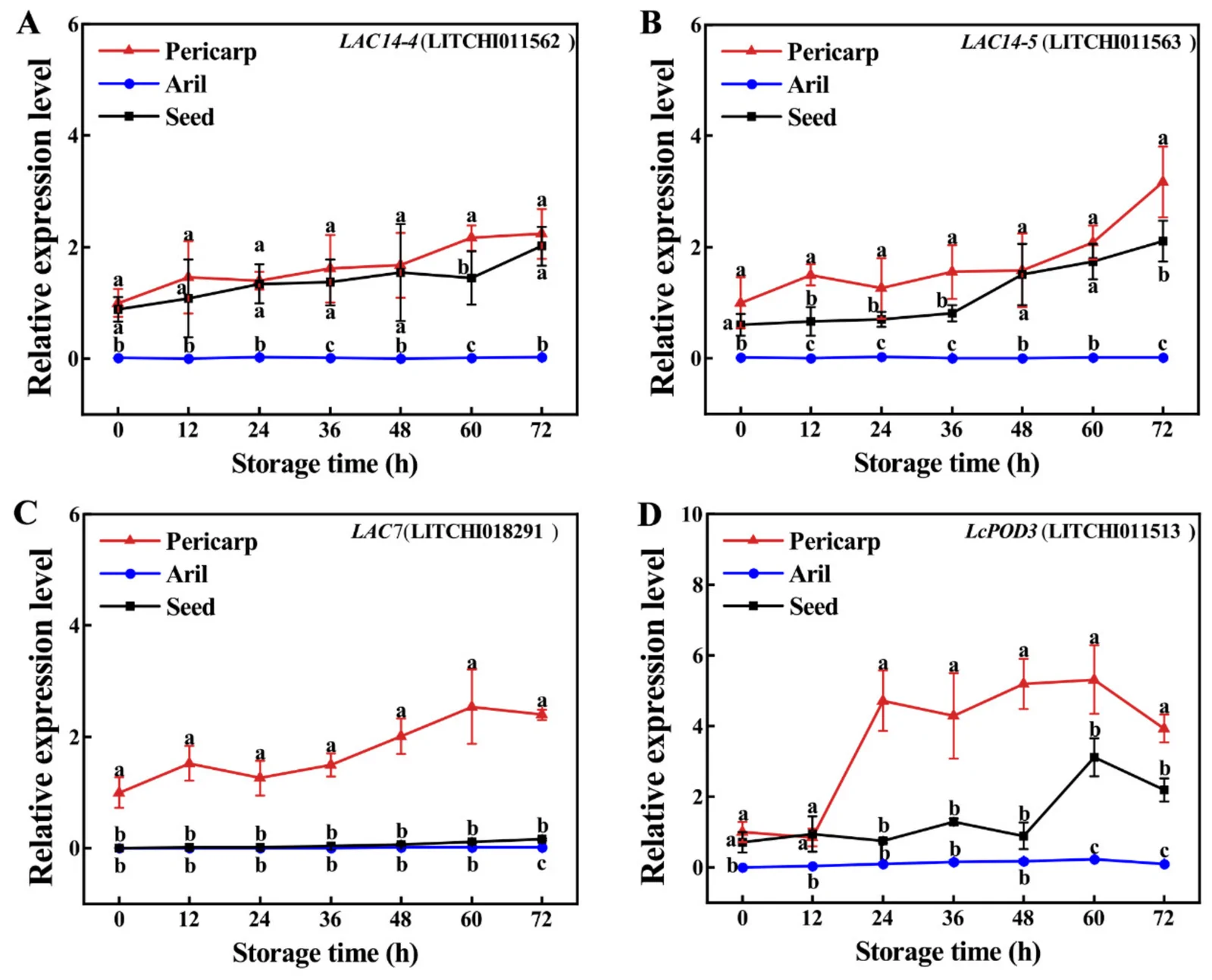
Relative expression of browning-related genes in different tissues of litchi fruit during storage. *LAC14-4* (LITCHI011562) (**A**), *LAC14-5* (LITCHI011563) (**B**), *LAC7* (LITCHI018291) (**C**), *LcPOD3* (LITCHI011513) (**D**). Data are means ± SE, *n* = 3. Different lowercase letters indicate significant differences among tissues at the same storage time (*p* < 0.05).

To further examine the expression patterns of browning-related genes, qRT-PCR was performed to analyze the relative expression levels of three LAC genes (*LAC14-4*, *LAC14-5*, and *LAC7*) and one *LcPOD3* gene in different tissues (Figure 7). The expression levels of these genes were generally higher in the pericarp and seed, with the pericarp showing higher levels than the seed, whereas they were relatively lower in the aril. In the pericarp, the expression of the three LAC genes increased progressively during storage. Specifically, the relative expression levels of *LAC14-4*, *LAC14-5*, and *LAC7* increased from approximately 1.0 at 0 h to 2.2, 3.2, and 2.4 at 72 h, corresponding to increases of approximately 120%, 220%, and 140%, respectively (Figure 7A–C). Their expression also increased in the seed, particularly for *LAC14-4* and *LAC14-5*, whereas *LAC7* expression remained low. In contrast, all three genes were expressed at consistently low levels in the aril. *LcPOD3* expression was high in the pericarp, low in the seed, and nearly absent in the aril (Figure 7D). *LcPOD3* expression in the pericarp increased from approximately 1.0 at 0 h to a peak of 5.4 at 60 h, before declining to 3.9 at 72 h, which was approximately fourfold higher than the initial level (Figure 7D). In the seed, *LcPOD3* expression increased from approximately 0.7 at 0 h to 2.2 at 72 h, whereas it remained low in the aril. The upregulation of browning-related genes in the pericarp and seeds was consistent with the enhancement of browning-related enzyme activities, further suggesting their potential involvement in browning of the pericarp and seeds.

## 4. Discussion

The tissue-specific browning observed in litchi fruit can be attributed to the unique anatomical structure of the fruit. The pericarp consists of three distinct layers—exocarp, mesocarp, and endocarp—whereas the edible aril develops from the funicle rather than from any portion of the pericarp [23]. Unlike typical fleshy fruits such as peach, where the edible flesh derives from the pericarp, the aril of litchi differs markedly from the pericarp in both tissue origin and function [4,7]. The pericarp serves protective roles, whereas the aril represents the fleshy edible tissue rich in nutrients. In addition, litchi seeds exhibit recalcitrant characteristics, including high moisture content and active metabolism, which may further influence postharvest behavior [7,24]. In the present study, the results were obtained from separated tissues; browning developed rapidly in the pericarp and on the cut surface of the seeds but not in the aril, confirming the tissue-specific nature of litchi postharvest browning (Figure 2). It is worth noting that seed browning was immediately induced by cutting, whereas pericarp browning developed progressively during storage. Pericarp browning, characterized by rising browning index and ΔE values and declining L*, a*, and b* values, was associated with continuous anthocyanin degradation and oxidative polymerization, which shifted the color from bright red to brown. The seed browned immediately upon cutting, with the browning index peaking rapidly yet color parameters continuing to shift progressively, despite the absence of detectable anthocyanins. In contrast, the aril remained stable throughout storage, with minimal changes in all colorimetric parameters and no detectable anthocyanins.

Membrane integrity is critical for maintaining normal cellular compartmentalization during postharvest storage. Membrane deterioration increases permeability and promotes REL, which is widely used as an indicator of membrane damage, with higher leakage reflecting more severe disruption [11]. In litchi, postharvest dehydration has been shown to aggravate pericarp membrane disruption and is associated with pericarp browning [2,25]. While membrane breakdown facilitates contact between phenolic substrates and oxidative enzymes, triggering enzymatic browning [26]. In the present study, REL and MDA contents increased with storage time across all three tissues, indicating progressive membrane damage and lipid peroxidation (Figure 3). The pericarp exhibited the highest REL and the largest increases, with pronounced MDA changes in both pericarp and seeds, whereas the aril showed minimal alterations. These findings suggest that the pericarp is particularly susceptible to storage stress, and that membrane deterioration was associated with or may contribute to the browning process, potentially by promoting contact between oxidative enzymes and phenolic substrates.

PAs are condensed tannins formed by the polymerization of flavan-3-ols and are important components of polyphenols and flavonoids. Previous studies have shown that litchi pericarp is rich in condensed tannins, EC, PC A2, and other PA-related components [27,28], and that PAs, along with other flavonoids and phenolic acids, are important constituents of phenolic compounds in the pericarp [29,30]. In pericarp, this trend is supported by previous studies on litchi pericarp phenolic metabolism. He et al. [31] reported, based on metabolomic and transcriptomic analyses, that EC and anthocyanins were major browning-related substrates that decreased during the browning stage of litchi pericarp. Deng et al. [32] further showed that EC, PC A2, and PC B2 were detected as major phenolic compounds in litchi pericarp and declined during storage. Zeng et al. [33] demonstrated that pericarp browning was correlated with PA and EC levels. Xu et al. [34] isolated and identified several A-type PAs and EC from litchi seeds, and Prasad et al. [35] also reported higher total phenolic content in the seeds than in the pericarp. Luo et al. [36] showed that metabolic changes were more pronounced in the litchi pericarp than in the aril through untargeted metabolomic analysis. In this study, the total phenolic and total flavonoid contents were significantly higher in the litchi pericarp and seeds than in the aril, indicating that these two tissues possess a richer pool of phenolic substrates (Figure 4). The major PA components in the pericarp and seeds were largely similar, HPLC results further showed that EC, PC A2, PC B1, and PC B2 were mainly localized in the pericarp and seeds, yet the two tissues exhibited different preferences for the accumulation of specific compounds. Both tissues showed a decline in PA content during storage, and their contents in these tissues progressively decreased over the storage period, whereas they were barely detected in the aril. Notably, the EC content in the pericarp was higher than that in the seed, whereas the seed contained abundant CT, which was not detectable in the pericarp (Figure 5). The distinct contribution of CT and EC to browning may be associated with the substrate specificity of oxidative enzymes. Previous studies demonstrated that the litchi LAC exhibited a strong preference for EC, whereas its catalytic activity toward CT was relatively limited [13,15]. In contrast, the enrichment of CT in seeds may reflect a different metabolic function. These findings further indicate that the pericarp is the major tissue basis for postharvest browning in litchi fruit.

Pericarp browning was associated with concerted changes in oxidative enzyme systems and phenolic substrates [37]. Previous studies have demonstrated that LAC and POD are key oxidative enzymes involved in litchi pericarp browning, as they can catalyze the oxidation of phenolic substrates, ultimately leading to the formation of polymeric brown pigments [15,38]. In recent years, increasing attention has been paid to the important role of LAC in litchi pericarp browning [15,37]. Fang et al. [13] found that LAC is involved in anthocyanin degradation in litchi pericarp, and subsequent studies further showed that LAC is closely associated with the oxidative polymerization of flavonoids [15]. In addition, analyses of the litchi LAC gene family indicated that several LAC members are closely related to polyphenol metabolism and postharvest pericarp browning [16]. In the present study, LAC and POD activities gradually increased (Figure 6), accompanied by the upregulation of the corresponding genes, including *LcLAC14-4/5*, *LcLAC7*, and *LcPOD3* (Figure 7), suggesting that the oxidative enzyme system was enhanced not only at the enzyme activity level and may also be regulated at the transcriptional level. Postharvest browning in litchi exhibits clear tissue specificity, with the pericarp being the primary site of browning due to its abundant phenolic substrates and oxidative enzymes, whereas the aril remains stable. The tissue-specific browning mechanisms also differ in their dependence on anthocyanins. Pericarp browning involves anthocyanin-mediated oxidative polymerization by LAC, which can polymerize anthocyanins with epicatechin (EC) and other PAs to form insoluble brown polymeric pigments [13,15]. In contrast, seed browning appears to be anthocyanin-independent, as the seed coat lacks detectable anthocyanin pigments. Instead, seed browning is primarily driven by mechanical damage (cutting-induced) that disrupts cellular compartmentalization, leading to direct oxidation of phenolic substrates (e.g., EC and PAs) by LAC and/or POD, without the involvement of anthocyanin intermediates. The present results were obtained from isolated tissues and may not fully reflect browning in intact fruit, as factors such as tissue integrity, intercellular communication, water status, and structural barriers in whole fruit may influence this process. Additionally, tissue separation may have accelerated browning; therefore, these findings require further validation in intact litchi fruit systems. These findings highlight the pericarp as the key target for preservation and future strategies may benefit from a multi-target approach that addresses these interconnected factors.

## 5. Conclusions

This study showed that postharvest browning in litchi fruit was highly tissue-specific, occurring mainly in the pericarp and on the cut surface of the seeds, whereas the aril showed no browning. Pericarp and seed browning was associated with enzymatic oxidation of phenolics, membrane damage, PA substrate depletion, and upregulated LAC/POD activities and LAC/POD-related gene expression. Both browned tissues contained higher levels of total phenolics, flavonoids, and PAs than the aril, with these phenolic substrates declining as browning progressed. The mechanisms underlying browning in the pericarp and seed differ considerably, as reflected by distinct PA profiles, along with markedly higher enzyme activities and gene expression levels. The phenolic profiles were tissue-specific: EC dominated in the pericarp, whereas CT was present only in the seed. LAC and POD activities, along with transcript levels of *LcLAC14-4/5*, *LcLAC7*, and *LcPOD3*, were markedly higher in the pericarp than in the seed and barely detectable in the aril. These findings indicate that pericarp browning is the key factor in postharvest quality deterioration of litchi fruit. Accordingly, delaying pericarp browning should serve as the primary focus of preservation strategies. Future research should investigate the biosynthetic regulation of tissue-specific PAs in pericarp and seeds. It should also be noted that the present results were obtained from separated tissues and require further validation in intact fruit systems during storage.

## Figures and Tables

**Table 1:** Two-way ANOVA of the effects of tissue type, storage time, and their interaction on physiological, biochemical, and molecular variables in isolated litchi tissues.

Variable	Tissue	Storage Time	Tissue × Storage Time
	F	F	F
L* value	401.575 **	24.639 **	6.724 **
a* value	2464.481 **	10.627 **	30.298 **
b* value	1818.283 **	1.624 ^ns^	4.065 **
Relative electrolyte leakage	219.264 **	59.630 **	20.303 **
Malondialdehyde content	902.973 **	180.577 **	12.844 **
Total phenolic content	5686.220 **	9.120 **	3.601 **
Total flavonoid content	3651.873 **	23.648 **	8.057 **
Epicatechin	2939.824 **	272.373 **	112.535 **
Procyanidin A2	3866.159 **	55.591 **	14.234 **
Procyanidin B1	55.087 **	7.990 **	3.243 **
Procyanidin B2	1640.534 **	44.426 **	22.200 **
LAC activity	606.054 **	29.888 **	15.605 **
POD activity	4056.631 **	93.621 **	52.596 **
*LAC14-4*	94.557 **	4.277 **	1.985 ^ns^
*LAC14-5*	152.600 **	13.451 **	5.371 **
*LAC7*	518.384 **	10.325 **	7.761 **
*LcPOD3*	296.830 **	28.556 **	15.745 **

Tissue types included the pericarp, aril, and seed, and storage times included 0, 12, 24, 36, 48, 60, and 72 h. Values represent the F statistics obtained from two-way ANOVA. The degrees of freedom for tissue type, storage time, and tissue × storage time were 2, 6, and 12, respectively. * *p* < 0.05; ** *p* < 0.01; ^ns^ indicates no significant effect at *p* ≥ 0.05.

## Data Availability

The original contributions presented in this study are included in the article/Supplementary Materials. Further inquiries can be directed to the corresponding authors.

## Supplementary Materials

The following supporting information can be downloaded at https://www.mdpi.com/article/10.3390/foods15172989/s1, Table S1: Primers used for qRT-PCR in this study.
