# Supplementary material for: Tissue-Specific Browning in Postharvest Litchi Fruit: Comparative Analysis of the Pericarp, Aril, and Seeds
Source: Foods. 2026 Aug 25;15(17):2989. doi: 10.3390/foods15172989 (PMC13565707; doi:10.3390/foods15172989)
Supplement: Supplementary file 1 [file foods-15-02989-s001.zip › foods-4484158-supplementary.pdf]

Supplementary Materials

Table S1. Primers used for qRT-PCR in this study

Gene name / ID	Primer name	Primer sequence (5′→3′)
Actin / LITCHI007623	Actin-F	ACCGTATGAGCAAGGAAATCACTG
	Actin-R	TCGTCGTACTCACCCTTTGAAATC
LcLAC14-4 / LITCHI011562	LAC14-4-F	ACCATTCGGCTTCATACGAC
	LAC14-4-R	CAACTTATCGCCACCCAGAT
LcLAC14-5 / LITCHI011563	LAC14-5-F	TGGTGGCATGCTCATAGTGA
	LAC14-5-R	CCACTGCCTCTTCATAGACT
LcLAC7 / LITCHI018291	LAC7-F	GTGTAGACGCAGAGTACACA
	LAC7-R	CGTAGACGATCATTCCTCTGG
LcPOD3 / LITCHI011513	POD-F	GCATCAGTCACTGTACCTCTTT
	POD-R	TCAATGGTAGGGCAGCTTTC
